# Aberrant functional connectivity in resting state networks of ADHD patients revealed by independent component analysis

**DOI:** 10.1186/s12868-020-00589-x

**Published:** 2020-09-18

**Authors:** Huayu Zhang, Yue Zhao, Weifang Cao, Dong Cui, Qing Jiao, Weizhao Lu, Hongyu Li, Jianfeng Qiu

**Affiliations:** 1 Shandong University of Science and Technology, No. 579, Qianwan’ gang Road, Qingdao, 266590 Shandong China; 2 Medical Engineering and Technology Research Center, Shandong First Medical University and Shandong Academy of Medical Sciences, Taian, 271016 China; 3 Radiology Department, Shandong First Medical University & Shandong Academy of Medical Sciences, Taian, 271016 China

**Keywords:** fMRI, RSNs, ADHD, Functional connectivity, ICA

## Abstract

**Background:**

ADHD is one of the most common psychiatric disorders in children and adolescents. Altered functional connectivity has been associated with ADHD symptoms. This study aimed to investigate abnormal changes in the functional connectivity of resting-state brain networks (RSNs) among adolescent patients with different subtypes of ADHD.

**Methods:**

The data were obtained from the ADHD-200 Global Competition, including fMRI data from 88 ADHD patients (56 patients of ADHD-Combined, ADHD-C and 32 patients of ADHD-Inattentive, ADHD-I) and 67 typically developing controls (TD-C). Group ICA was utilized to research aberrant brain functional connectivity within the different subtypes of ADHD.

**Results:**

In comparison with the TD-C group, the ADHD-C group showed clusters of decreased functional connectivity in the left inferior occipital gyrus (p = 0.0041) and right superior occipital gyrus (p = 0.0011) of the dorsal attention network (DAN), supplementary motor area (p = 0.0036) of the executive control network (ECN), left supramarginal gyrus (p = 0.0081) of the salience network (SN), middle temporal gyrus (p = 0.0041), and superior medial frontal gyrus (p = 0.0055) of the default mode network (DMN), while the ADHD-I group showed decreased functional connectivity in the right superior parietal gyrus (p = 0.0017) of the DAN and left middle temporal gyrus (p = 0.0105) of the DMN. In comparison with the ADHD-I group, the ADHD-C group showed decreased functional connectivity in the superior temporal gyrus (p = 0.0062) of the AN, inferior temporal gyrus (p = 0.0016) of the DAN, and the dorsolateral superior frontal gyrus (p = 0.0082) of the DMN. All the clusters surviving at p < 0.05 (AlphaSim correction).

**Conclusion:**

The results suggested that decreased functional connectivity within the DMN and DAN was responsible, at least in part, for the symptom of inattention in ADHD-I patients. Similarly, we believed that the impaired functional connectivity within networks may contribute to the manifestations of ADHD-C patients, including inattention, hyperactivity/impulsivity, and unconscious movements.

## Background

Attention-deficit/hyperactivity disorder (ADHD) has emerged as a common contributor to neuro developmental disorders as well as frequent psychological and behavioral problems among children [1]. The global prevalence of ADHD is about 5.29%. According to the previous studies, any variation in the prevalence estimates can be attributed to the methodological characteristics of different studies instead of discrepancies in the actual distribution of ADHD [2]. There are a great number of adolescent ADHD patients in the world; thus, management and treatment of ADHD patients is very important. ADHD is mainly characterized by symptoms of inattention, impulsivity, and hyperactivity. Diagnosis of ADHD is mainly depended on the levels of symptoms listed in DSM-IV [3] and is usually conducted by parents or teachers, which is subjective. Typically, ADHD can be categorized into three subtypes: hyperactive-impulsivity (ADHD-HI), persistent inattention (ADHD-I), and a combination of both (ADHD-C) [4, 5]. This disorder is often accompanied by learning difficulties or conduct disorders [6, 7], which can greatly affect the interpersonal skills and academic performance of the patients. Many studies have pointed out that the subjective diagnoses make it difficult to draw a line between the normal level and the level of ADHD symptoms that need treatment [8]. Thus, studies on objective diagnosis of ADHD are of great significance, and research on ADHD has become a major topic of interest in medicine and psychology in recent years.

In previous studies, resting-state functional MRI (rs-fMRI) was widely used to examine the brain of ADHD patients [9, 10]. In rs-fMRI studies of brain function, abnormalities were found in the prefrontal cortex, anterior cingulate cortex, putamen, temporal cortex, and cerebellum [11, 12]. rs-fMRI has become a research hotspot that is being increasingly used to achieve obvious results in many fields, such as neuroscience, spiritual science, biological science, and statistics, and it has been shown to be helpful for the diagnosis and treatment of ADHD [13, 14]. A growing body of literature shows that communication abnormalities among and within neural networks may underlie ADHD [15]. rs-fMRI can effectively identify such network abnormalities, and it is unconstrained by limitations yet reliable for this purpose. In rs-fMRI experiments, subjects are awake and are asked to simply rest while lying in the MRI scanner, so brain activity can be considered “spontaneous” rather than stimulus- or task-driven. As previously mentioned, most researchers focused on the default mode network (DMN), while less attention was paid to other brain networks or differences between the two types of ADHD. Therefore, we speculated that the auditory network (AN), dorsal attention network (DAN), executive control network (ECN), salience network (SN), and sensorimotor network (SMN) are also related to ADHD, and we compared the differences in the functional connectivity (FC) of six resting-state brain networks (RSNs) between two ADHD subtypes.

In the present study, group independent component analysis (ICA), a data-driven approach, was adopted to extract the components [16]. ICA is a widely used method for statistical analysis of fMRI data [17, 18]. Without any prior information, this method can effectively determine the functional characteristics of mutually correlated brain components [19]. We hope to identify the differences in these RSNs among patients with different subtypes of ADHD by comparing the FCs of the six RSNs among the three groups. We speculate that the symptoms of ADHD patients are related to abnormal FCs of these RSNs.

## Materials and methods

### Subjects

Public fMRI data were downloaded from the ADHD-200 Global Competition (http://fcon1000.projects.nitrc.org/indi/adhd200/index.html) and selected exclusively from the New York University (NYU) Child Study Center. In accordance with HIPAA guidelines and 1000 Functional Connectomes Project protocols, all datasets are anonymous, with no protected health information included. For both ADHD and TD subjects, the inclusion criteria were as follows: age of 7–17 years, no history of neurological disease, and no diagnosis of either schizophrenia or affective disorder, an image covering at least 95% of the brain, an IQ score > 80, and head movement less than 2.0. Subjects were enrolled if they were right-handed and their information was complete (e.g., age, Verbal IQ, or Performance IQ). Finally, fMRI data from a total of 155 volunteers aged between 7 and 17 years were collected, including 67 typically developing controls (TD-C), 56 ADHD-C patients, and 32 ADHD-I patients (the number of ADHD-HI patients was too small to be studied). IBM SPSS software (Armonk, NY, v. 22.0) was used for statistical analysis. One-way analysis of variance was performed with age, ADHD index, verbal IQ, performance IQ, and Full IQ, and the Chi squared test was used to evaluate the differences in gender among the three groups. A *p* value of < 0.05 was considered statistically significant, as shown in Table 1. The symptoms of ADHD were assessed using the Conners Parent Rating Scale-Revised, Long version (CPRS-LV) [20].

**Table 1:** Demographic characteristics of the samples

	TD-C (n = 67)	AC (n = 56)	AI (n = 32)	F/χ^2^ value	p value	Post hoc
Age	12.10 ± 2.92	10.98 ± 2.57	12.30 ± 2.67	3.400^a^	0.036	NA
Gender (Female)	67 (30)	56 (45)	32 (20)	17.147^b^	< 0.001	TD-C > AC = AI
ADHD index	45.59 ± 6.52	71.64 ± 9.03	70.06 ± 9.34	188.334^a^	< 0.001	AC = AI > TD-C
Verbal IQ	112.13 ± 13.92	108.79 ± 13.23	107.88 ± 19.07	1.285^a^	0.280	NA
Performance IQ	106.88 ± 14.18	102.95 ± 14.09	106.75 ± 14.98	1.323^a^	0.269	NA
Full-Scale IQ	110.75 ± 14.01	106.79 ± 13.68	108.44 ± 15.97	1.183^a^	0.309	NA

The data (except Gender) were shown in mean ± standard deviation, the Gender was shown in total (number of females)

*AC* ADHD-Combined patients, *AI* ADHD-Inattentive patients, *IQ* Intelligence Quotient, *NA* not applicabl, *TD-C* Typically-Developing Controls

^a^one-way analysis of variance

^b^Chi square test

The fMRI data were acquired using a single-shot echo-planar imaging (GRE-EPI) sequence with the following imaging parameters: repetition time (TR) = 2000 ms; echo time (TE) = 15 ms; flip angle (FA) = 90°; FOV reading = 240 mm; slice thickness = 4 mm; number of slices = 33; and voxel size = 3 × 3 × 4 mm^3^, time points = 176, and acquisition matrix = 80 × 80.

### Data preprocessing

The original fMRI data were preprocessed using a public toolbox named DPABI (for Data Processing & Analysis of Brain Imaging, http://rfmri.org/dpabi). The preprocessing steps were as follows: (1) remove the first 10 volumes to ensure that the BOLD signal was stable; (2) slice timing, correct the difference due to acquisition times between slices in the volume; (3) head motion correction; (4) normalization, register the data to the EPI standard template and resample it to 3.0 × 3.0 × 3.0 mm^3^; and (5) spatial smoothing with a 6-mm full width at half maximum (FWHM) Gaussian kernel [21]. Subjects whose head movement exceeded 2.0 mm were excluded.

### ICA and determination of RSNs

We employed the ICA, a data-driven analysis technique [22], to identify the RSNs of the ADHD patients and TD-C. The ICA approach had been applied to simulation data by the research group of Dr. Calhoun [5, 23, 24]. In our study, a group ICA analysis was conducted to decompose the data into ICs using GIFT toolbox (Version 4.0, https://trendscenter.org/software/gift/) for all subjects, including ADHD patients and TD-C. This toolbox implements estimation of the independent components (ICs) on concatenated data of subject-specific spatial maps and time courses across subjects [25]. First, The principal component analysis was used to reduce the data dimensionality. We used the minimum description length (MDL) criteria to define the number of ICs, indicating 20 ICs for our functional dataset, which is sufficient to capture the most frequently observed large-scale resting-state networks [26]. The infomax algorithm that was repeated 20 times in ICASSO, was then used as an independent component estimation. The resulting components were clustered to estimate the reliability of the decomposition. Finally, the individual time courses and spatial maps were reconstructed using the group ICA back-reconstruction method.

The RSN components were subsequently selected via an automated process that defines the components that most closely matched the RSNs for each individual subject, based on spatial correlation analyses with the RSNs templates. All templates represent regions that have repeatedly been implicated in the RSNs.

**Fig. 1:**
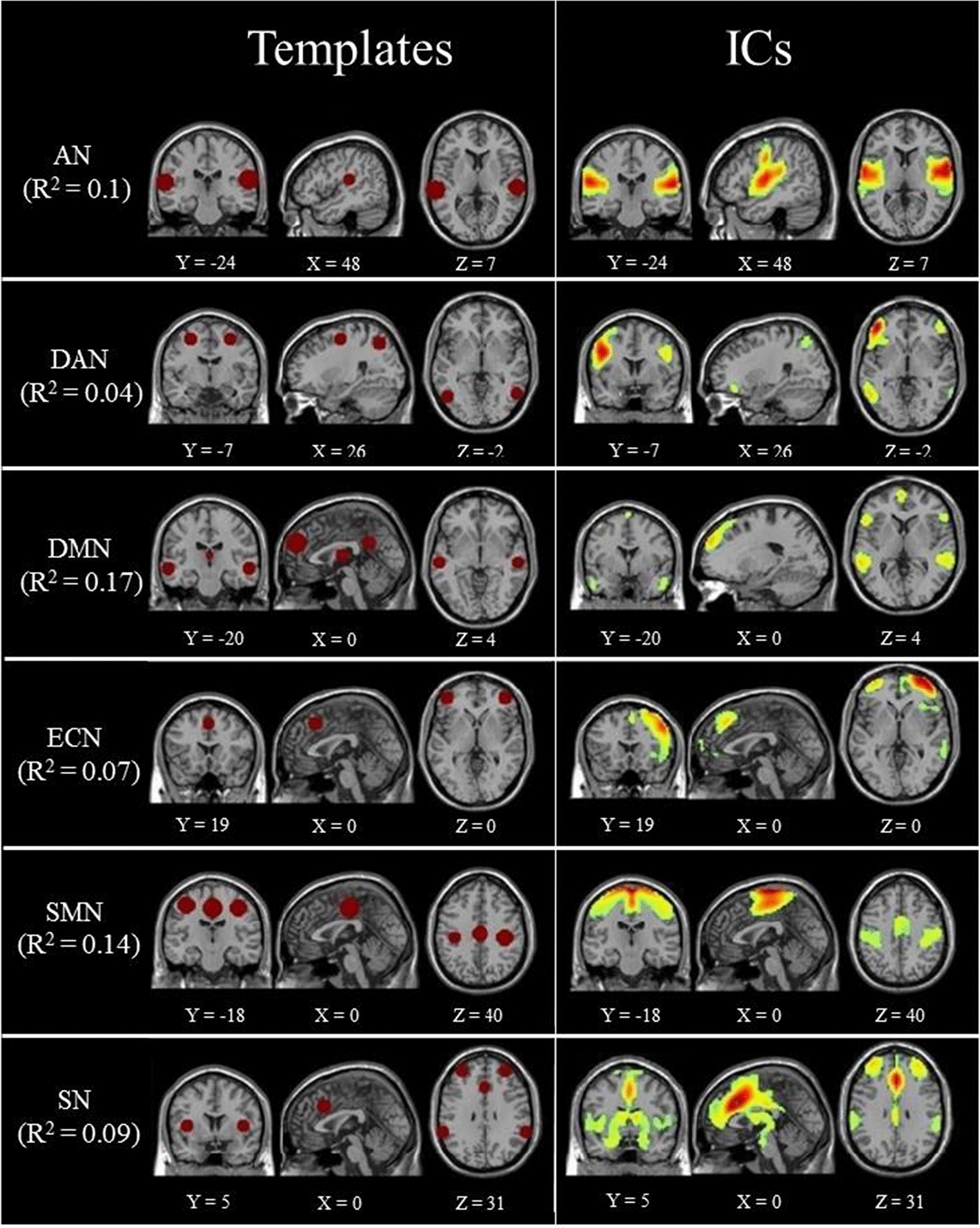
The results of spatial correlation for ICs and templates. R^2^ refers to the spatial correlation coefficient between the ICs and the templates. AN: auditory network; DAN: dorsal attention network; DMN: default mode network; ECN: executive control network; SMN: sensorimotor network; SN: salience network

**Fig. 2:**
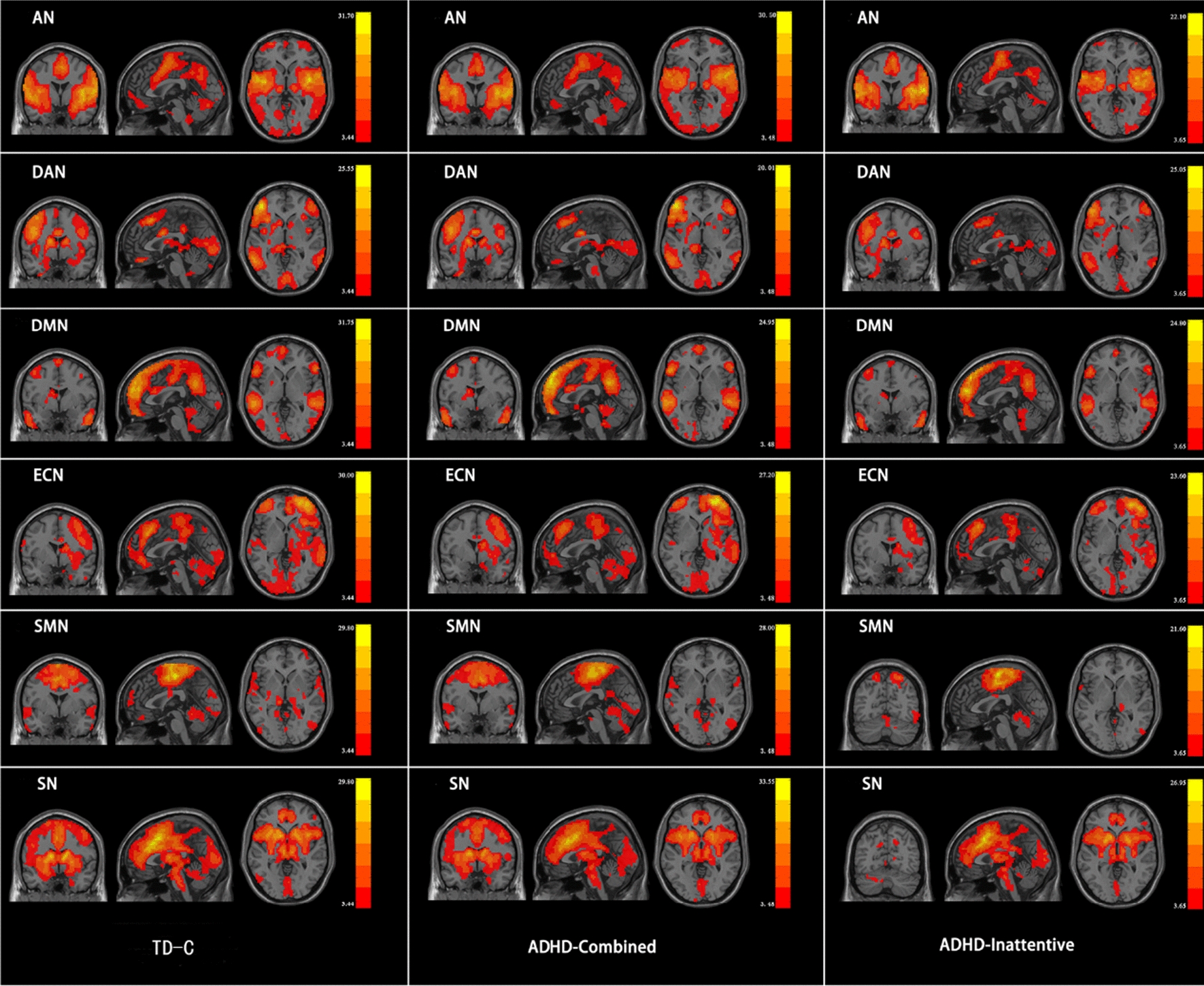
Six components obtained from all subjects and the corresponding resting state networks. Color bar indicates the t-value. AN: auditory network. DAN: dorsal attention network. DMN: default mode network. ECN: executive control network. SMN: sensorimotor network. SN: salience network

There were six RSNs of interest, namely, the AN, DAN, DMN, ECN, SN, and SMN. All RSN templates were created with WFU_PickAtlas (https://www.nitrc.org/projects/wfu_pickatlas/) in the SPM toolbox based on centroid coordinates and radii. The component with the largest correlation coefficient was selected as the RSN we were interested in. A total of six components were identified. Figure 1 shows the templates of the six RSNs and the selected corresponding ICs that had the highest spatial correlation with the template. In order to verify whether the six ICs of each group were zero, a one-sample t-test was performed and according to the settings in the previous study [27], we set a threshold of p < 0.05, as shown in Fig. 2.

### Statistical analysis

On the basis of the number of original subjects, the selected ICs (z-score value) were reclassified into three groups. The Resting-State fMRI Data Analysis Toolkit plus V1.2 (RESTplus V1.2, http://restfmri.net/forum/RESTplusV1.2) was the toolbox of our choice for statistical analysis. In order to verify whether the six ICs differed among the three groups, analysis of variance (ANOVA) on ICs (AlphaSim correction, p < 0.05) was performed with the result of the one-sample t-test serving as an explicit mask, where age and gender were controlled as covariates. The differences between the groups of ICs were obtained by two-sample t-test (AlphaSim correction, p < 0.05, cluster > 10) with the result of ANOVA serving as an explicit mask. Similarly, age and gender were eliminated as covariates. Finally, a Spearman correlation analysis was performed between the ADHD index and mean signals of ICs that we delineated as ROIs.

## Results

### ANOVA showed differences between the three groups

**Fig. 3:**
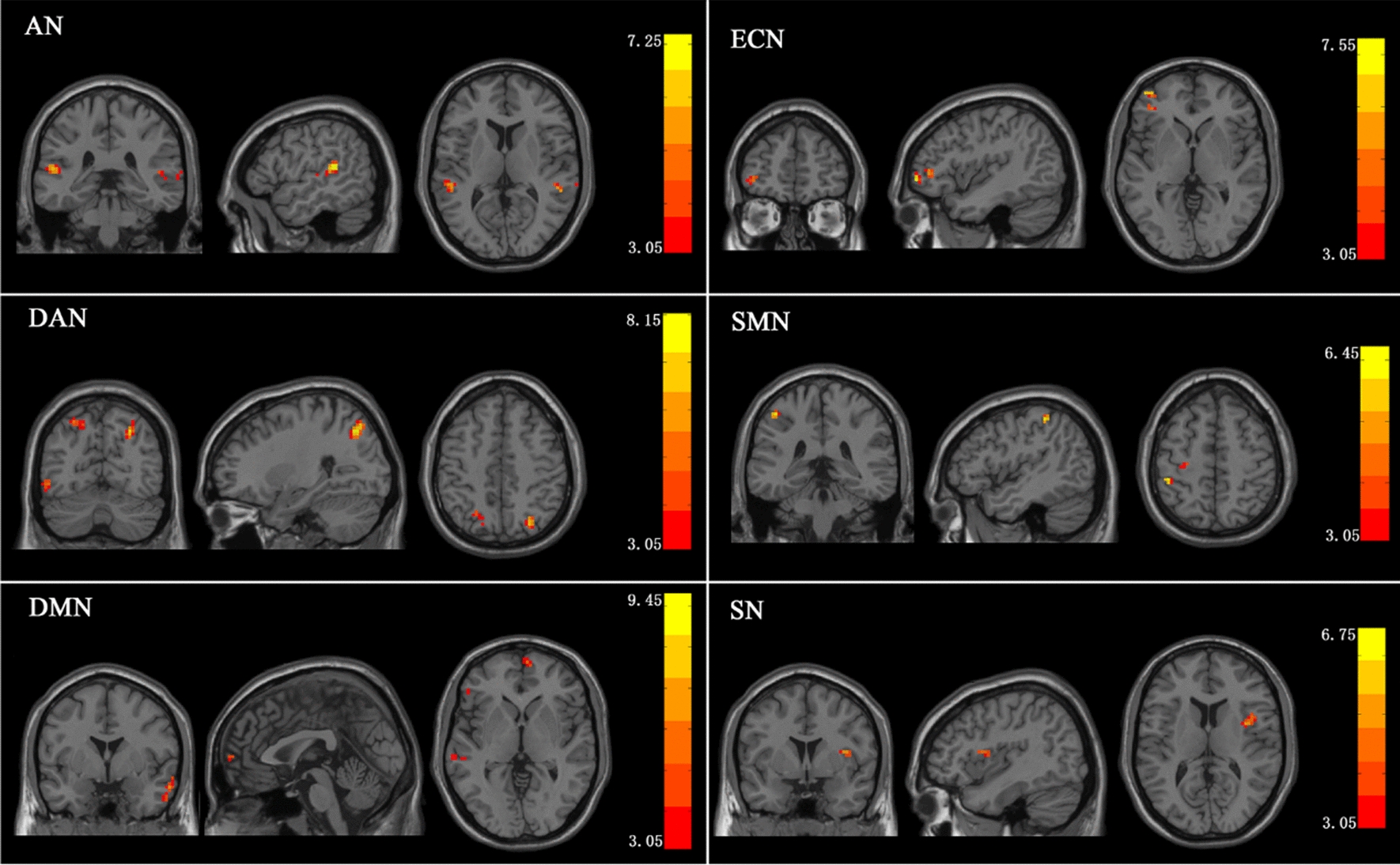
The results of ANOVA. Color bar indicates the F-value; AN: auditory network. DAN: dorsal attention network. DMN: default mode network. ECN: executive control network. SMN: sensorimotor network.SN: salience network

The ANOVA results for all ICs are shown in Fig. 3 and Table 2, indicating the brain regions where there may be differences between the six ICs in the three groups of subjects. The regions depicted in red-yellow shown in Fig. 3 indicate differences in RSNs between the three groups. The F values and p values of the most significant differences in each RSN are shown in Table 2. As shown in Fig. 3, brain regions with significant differences appeared in the superior temporal gyrus of the AN, superior parietal gyrus and occipital lobe of the DAN, middle temporal gyrus and superior medial frontal gyrus of the DMN, supplementary motor area of the ECN, precentral gyrus of the SMN, as well as supramarginal gyrus of the SN.

**Table 2:** Analysis of Variance for all ICs

RSNs	F-value	p-value
AN	7.9462	0.006
ECN	6.8647	0.010
DMN	9.5813	0.002
DAN	16.6126	0.001
SMN	5.8913	0.016
SN	6.3573	0.013

*AN* auditory network, *DAN* dorsal attention network, *ECN* executive control network, *SMN* sensorimotor network, *SN* salience network

### Comparisons between groups

The results of the two-sample t-test are presented in Table 3, which shows the differences between the groups of 6 ICs.

**Table 3:** Regions exhibiting altered functional connectivity in ADHD patients

	Voxels	MNI			t	p	Region (AAL)
		x	y	z			
AC < TD-C							
DAN	30	− 51	− 72	− 6	− 2.687	0.0041	Inferior occipital gyrus
DAN	28	27	− 69	45	− 3.139	0.0011	Superior occipital gyrus
ECN	10	− 3	21	45	− 2.730	0.0036	Supplementary motor area
SN	13	− 54	− 39	30	− 2.436	0.0081	Supramarginal gyrus
DMN	59	− 57	− 30	− 3	− 2.687	0.0041	Middle temporal gyrus
DMN	59	− 12	60	27	− 2.583	0.0055	Superior medial frontal gyrus
AI < TD-C							
DAN	28	27	− 72	51	− 3.002	0.0017	Superior parietal gyrus
DMN	59	− 66	− 27	− 3	− 2.347	0.0105	Middle temporal gyrus
AI > TD-C							
AN	15	− 57	− 24	21	2.845	0.0027	Supramarginal gyrus
SMN	17	− 33	− 21	57	2.893	0.0024	Precentral gyrus
DMN	59	9	54	21	1.958	0.0265	Superior medial frontal gyrus
AC < AI							
AN	29	− 51	− 36	15	− 2.550	0.0062	Superior temporal gyrus
DAN	44	− 48	− 60	− 6	− 3.023	0.0016	Inferior temporal gyrus
ECN	12	− 9	24	48	− 2.986	0.0018	Superior frontal gyrus
DMN	59	− 15	51	24	− 2.450	0.0082	Superior frontal gyrus
AC > AI							
DMN	59	− 63	− 24	− 9	2.627	0.0051	Middle temporal gyrus

*TD-C* Typically-Developing Controls, *AC* ADHD-Combined patients, *AI* ADHD-Inattentive patients, *AN* auditory network, *DAN* dorsal attention network, *ECN* executive control network, *SMN* sensorimotor network, *SN* salience network

#### The differences between ADHD-C and TD-C

**Fig. 4:**
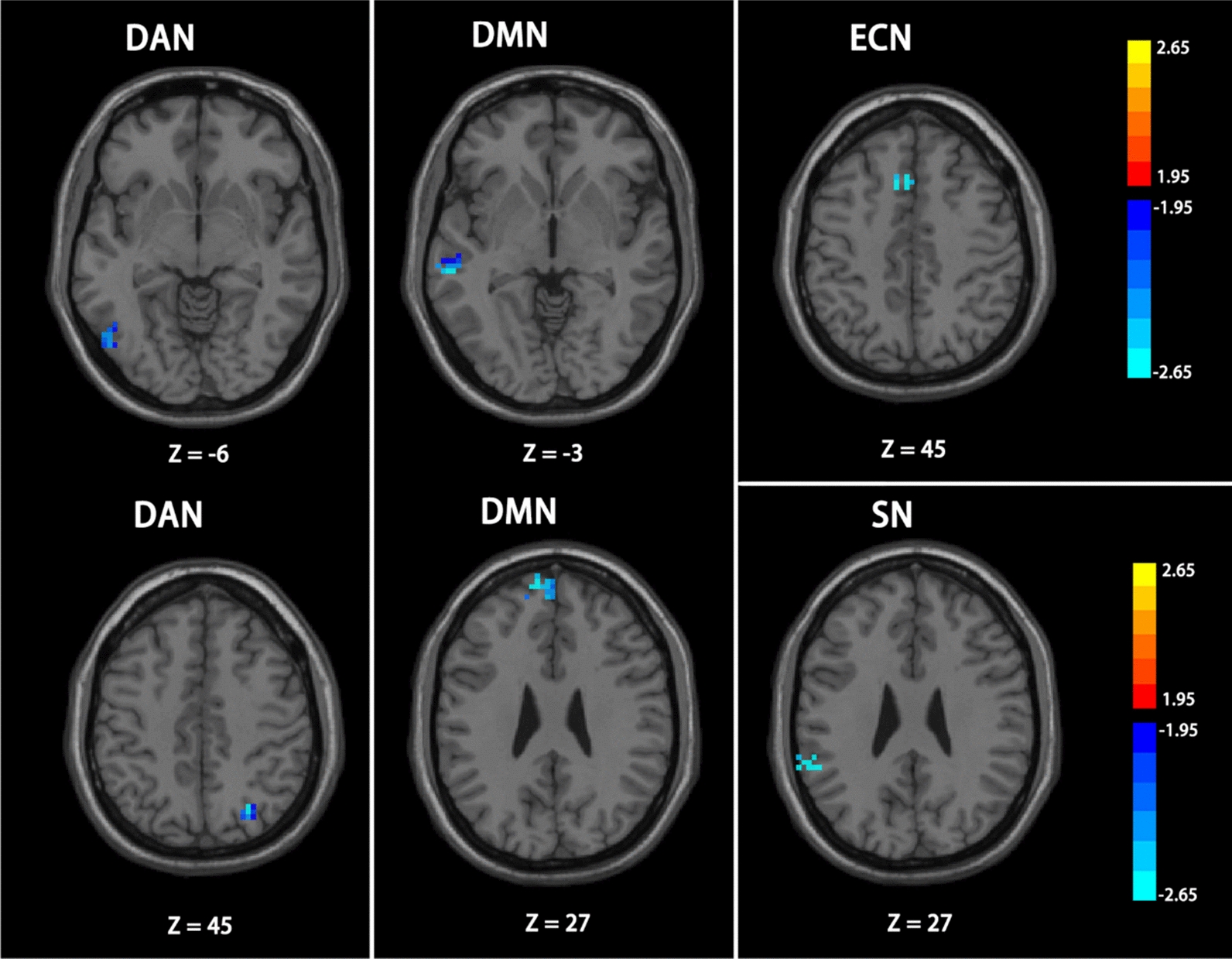
The difference between ADHD-C patients and healthy controls. Color bar indicates the t-value; Significant clusters are depicted in red-yellow at a threshold of p < 0.05. The clusters depicted in blue shows reduced functional connectivity

In comparison with the TD-C group, the ADHD-C group showed decreased FC in the DAN, ECN, SN, and DMN (shown in Fig. 4). The clusters of weaker connectivity were located in the left inferior occipital gyrus (p = 0.0041) and right superior occipital gyrus (p = 0.0011) of DAN, supplementary motor area (p = 0.0036) of ECN, left supramarginal gyrus (p = 0.0081) of SN, and middle temporal gyrus (p = 0.0041) and superior medial frontal gyrus (p = 0.0055) of DMN, with the differences for all areas at p < 0.05 (AlphaSim correction). However, RSNs with considerably enhanced FC were not observed in ADHD-C.

#### The differences between ADHD-I and TD-C

**Fig. 5:**
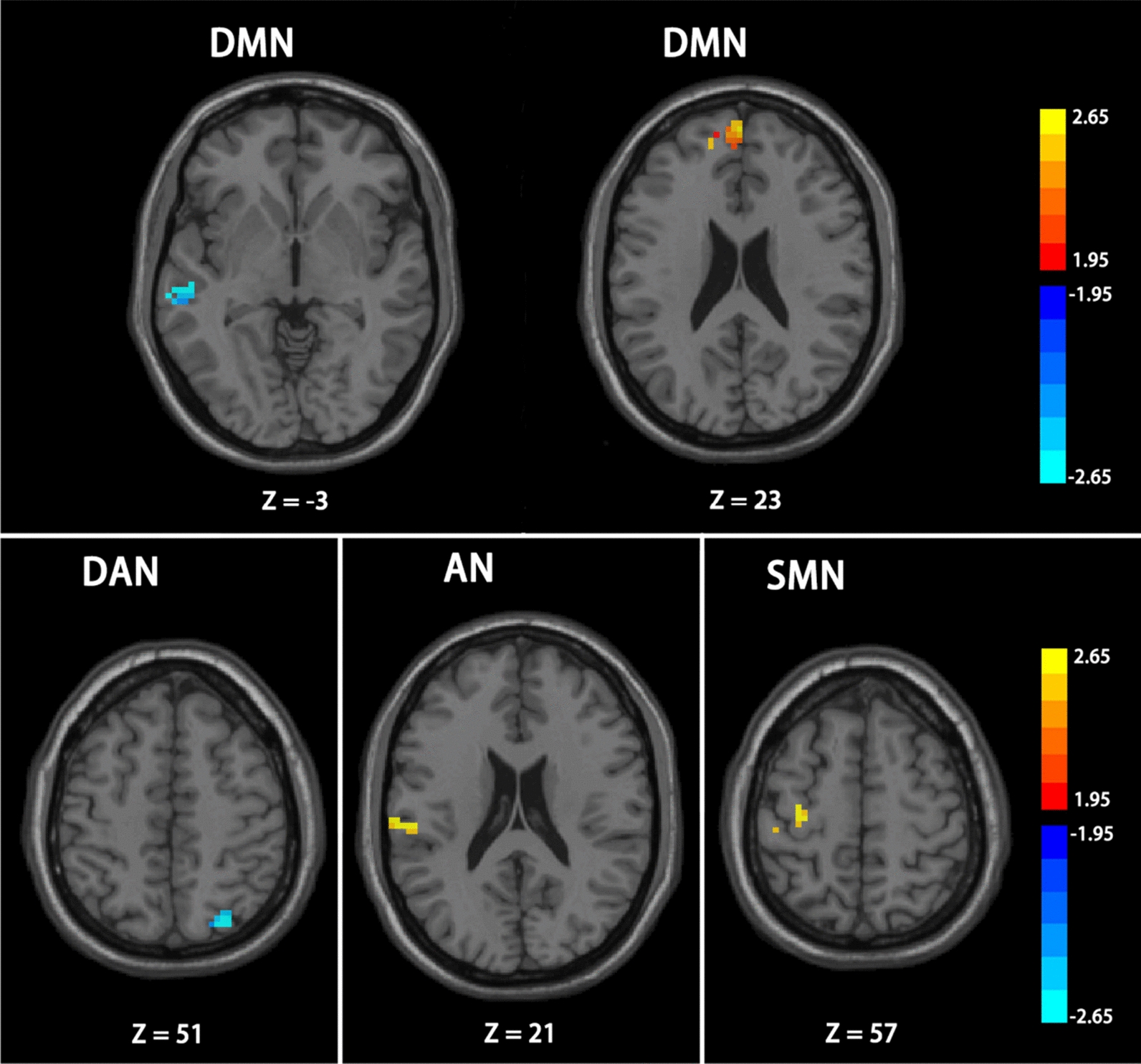
The differences between ADHD-I patients and healthy controls. Color bar indicates the t-value; Significant clusters are depicted in red-yellow at a threshold of p < 0.05. The clusters depicted in red (DMN (right), AN and SMN) shows the enhanced functional connectivity. And the reduced functional connectivity in DAN and DMN (left) is depicted in blue

Figure 5 indicates that ADHD-I patients showed decreased FC in comparison to the TD-C group in a few areas, namely, the right superior parietal gyrus (p = 0.0017) of DAN and the left middle temporal gyrus (p = 0.0105) of DMN. In contrast, stronger FC was observed within three RSNs, including the supramarginal gyrus (p = 0.0027) of AN, the precentral gyrus (p = 0.0024) of SMN, and the medial frontal gyrus (p = 0.0265) of DMN.

#### The differences between ADHD-C and ADHD-I

**Fig. 6:**
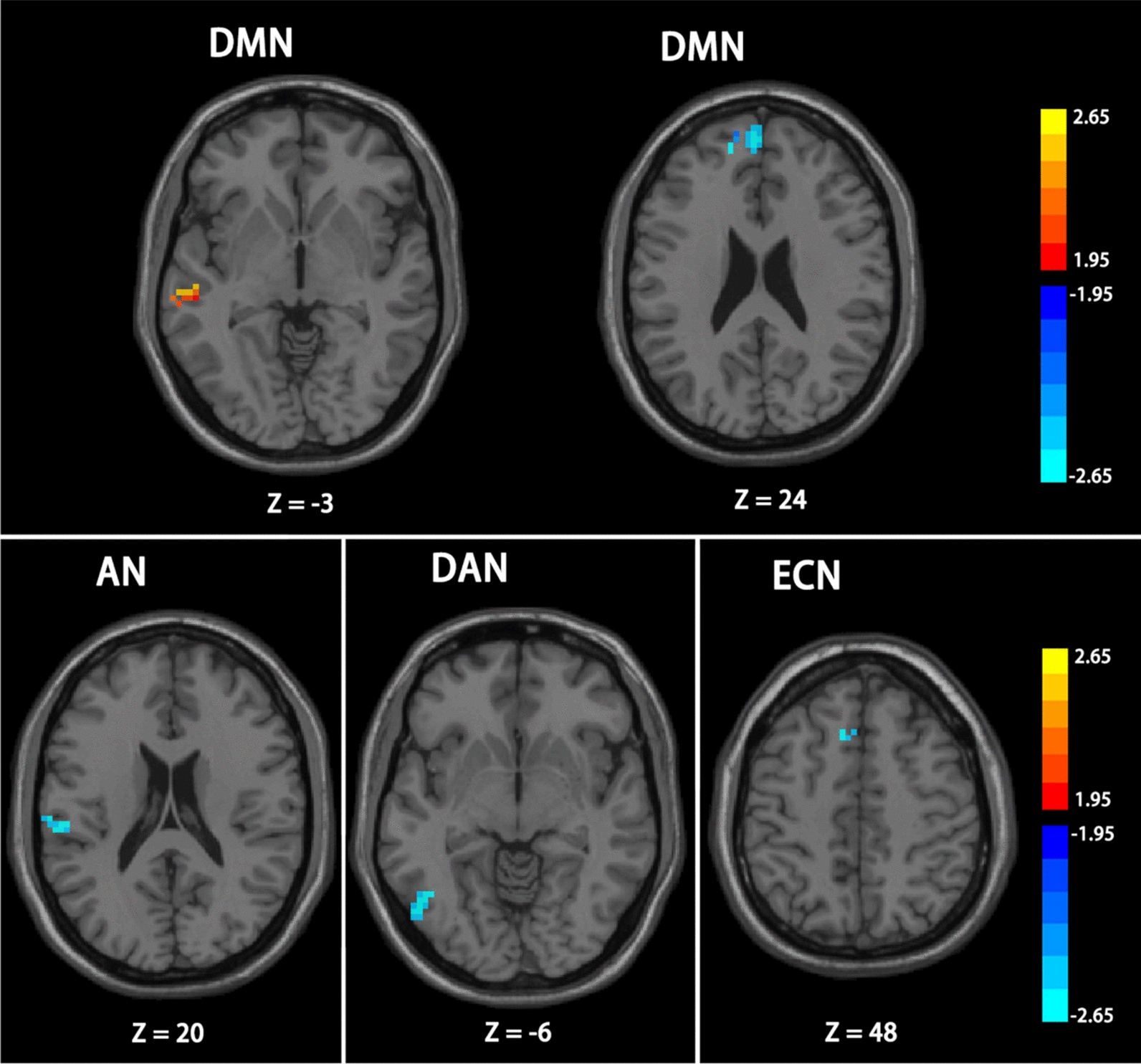
The difference between ADHD-C patients and ADHD-I patients. Color bar indicates the t-value; Significant clusters are depicted in red-yellow at a threshold of p < 0.05 The clusters depicted in blue (AN, DAN, ECN and DMN (right)) shows the reduced functional connectivity. And the enhanced functional connectivity in DMN (left) is depicted in red

In comparison with the ADHD-I group, the ADHD-C group showed weaker FC mainly in the superior temporal gyrus (p = 0.0062) of AN, inferior temporal gyrus (p = 0.0016) of DAN, as well as dorsolateral superior frontal gyrus (p = 0.0082) of DMN. However, an increase in the FC was found in the middle temporal gyrus (p = 0.0051) of DMN, as illustrated in Fig. 6.

### Correlation analysis

The above results have demonstrated a significant area of FC abnormalities in RSNs in ADHD patients. A Spearman correlation analysis was performed between ADHD index and the mean signals of ICs that we delineated as ROIs, in order to verify whether the ADHD indices of the two groups of ADHD patients were associated with FC abnormalities. All correlation analyses were performed in the ADHD-C and ADHD-I groups. An inverse relationship with the ADHD index was only found for the left supplementary motor area of ECN in the ADHD-C group (r = − 0.267, p = 0.047). The results showed no significant difference (p > 0.05) after a multiple comparison correction.

## Discussion

In this study, group ICA was performed and both subtypes of ADHD patients showed impaired FCs in major RSNs in comparison with TD-C. We found that the FCs of the DAN, ECN, SN, and DMN were significantly reduced in ADHD-C patients, including the right inferior occipital gyrus and the superior occipital gyrus of DAN, supplementary motor area of ECN, left supramarginal gyrus of SN, and middle temporal gyrus and superior medial frontal gyrus of DMN.

Many previous studies have demonstrated abnormal functional connections within the DMN of ADHD patients, especially the temporal lobe [25, 28, 29]. DMN is a commonly used brain network in fMRI studies and is considered to be associated with a wide range of neuropsychiatric diseases [30]. In our study, in comparison with the TD-C group, both subtypes of ADHD patients showed reduced FC in the occipital lobe of DAN and the middle temporal gyrus of DMN. However, in the superior medial frontal gyrus of DMN, the two groups performed inversely. Although DMN and DAN activity were believed to show opposite trends [31, 32], Matthew L. Dixon demonstrated that there is no anticorrelation between some subsystems and DMN [33]. These could explain our findings that reduced FC occurred simultaneously in DAN and DMN. We also observed differences in FCs between the two groups of ADHD subtypes. Patients with ADHD-C showed more reduced FC, such as the inferior temporal gyrus of DAN and superior frontal gyrus of DMN. Only a portion of the middle temporal gyrus of DMN has shown an enhanced FC. One explanation for the difference may be the diverse clinical symptoms between the two subtypes of ADHD patients. DMN shows greater activation during periods of silence or in a resting state [30]. In contrast, DAN is routinely activated during attention-demanding cognitive tasks [34]. Thus, we speculated that abnormal changes in FC exhibited by DMN and DAN may be related to the attention deficit in ADHD patients, which has been proven by some previous studies [35, 36].

When comparing the ADHD-I to the TD-C group, we found a few regions showing enhanced FC, such as the supramarginal gyrus of AN and the precentral gyrus of SMN. Previous studies have shown that patients with ADHD were more sensitive to sound, which may be related to the enhanced FC in AN [5]. Jean-Arthur reported that ADHD patients had perceptual inundation [37]. To the best of our knowledge, few studies have focused on the changes in AN and SMN, and few voxels in these regions were found in our study, which may be due to differences in sample size. Thus, we have not provided additional discussions about these regions.

Daniel von Rhein revealed that SN plays a role in supervision and decision when the brain processes external stimuli [27]. The main functions of the SN are to integrate information from different modalities such as sensory information and bodily states in order to establish goal-directed behavior and to process emotion-related information. Our findings showing reduced FC of the SN in ADHD-C patients confirmed the conclusions proposed by Daniel von Rhein [27]. In addition, the ECN has been shown to participate in multiple advanced cognitive tasks and play an important role in adaptive cognitive control [38]. The decreased FC in ECN may explain why ADHD-C patients fail to control their emotions and exhibit impulsive aggression or other conduct disorders [39, 40]. Two subtypes of ADHD patients also differ in FC of ECN. In the ADHD-C group, the superior frontal gyrus of ECN showed weaker FC. We speculate that this change may explain why ADHD-C patients are more hyperactive than ADHD-I patients.

## Conclusions

In conclusion, group ICA allowed the use of interrelated analytical methods for evaluating FCs of ADHD in adolescents in this study. We found that these major RSNs in both subtypes of ADHD patients showed FC changes in comparison with the TD-C group, and FC differences were also observed between the two subtypes of ADHD patients. Our study elucidates the abnormal changes in the RSNs of different subtypes of adolescent ADHD patients, which may prove helpful for the management of adolescent ADHD patients. In the future, studies employing a multimodal imaging approach using techniques such as structural MRI, diffusion tensor imaging and fMRI, will be necessary to comprehensively investigate the brain changes in adolescents with ADHD. These further studies will contribute to the management and treatment of ADHD in adolescents.

## Limitations

One of the limitations of our study was the absence of any psychological assessment of the cognitive parameters associated with ADHD. Moreover, the participant numbers in the three groups were substantially different, which was likely to have an impact on the results of statistical analysis. In addition, the six RSN templates were created by WFU_PickAtlas in the SPM toolbox directory based on centroid coordinates and radii. The differences between our templates and the actual anatomical RSNs would have also affected the accuracy of our results.

## Data Availability

The datasets analysed 329 during the current study are available in the [ADHD-200 Global Competition] repository, data usage is unrestricted for non-commercial research purposes. As per INDI protocol, the datasets simply require that user register with the NITRC and 1000 Functional Connectomes Project to gain access. [http://fcon_1000.projects.nitrc.org/indi/adhd200].

## Abbreviations

ADHD: Attention-deficit/hyperactivity disorder
ADHD-C: ADHD-Combined
ADHD-HI: ADHD-Hyperactive-impulsivity
ADHD-I: ADHD-Inattentive
AN: Auditory network
DAN: Dorsal attention network
DMN: Default mode network
DSM-IV: The fourth edition of the Diagnostic and Statistical Manual of Mental Disorders
ECN: Executive control network
FA: Flip angle
FC: Functional connectivity
FOV: Field of view
HIPAA: Health Insurance Portability and Accountability Act
ICA: Independent component analysis
MRI: Magnetic resonance imaging
ROI: Region of interest
rs-fMRI: Resting-state functional MRI
RSNs: Resting-state brain networks
SMN: Sensorimotor network
SN: Salience network
TD-C: Typically-developing controls
TE: Echo time
TR: Repetition time
